# Synthesis of wollastonite from AlF_3_-rich silica gel and its hardening in the CO_2_ atmosphere

**DOI:** 10.1038/s41598-019-54219-6

**Published:** 2019-12-02

**Authors:** Andrius Gineika, Raimundas Siauciunas, Kestutis Baltakys

**Affiliations:** Department of Silicate Technology, Kaunas University of Technology, Radvilenu pl. 19, LT–50270 Kaunas, Lithuania

**Keywords:** Chemistry, Environmental chemistry, Green chemistry, Inorganic chemistry, Materials chemistry, Physical chemistry, Process chemistry, Chemical synthesis, Environmental sciences, Environmental chemistry

## Abstract

This work combines some aspects of eco-friendliness: consumption of toxic waste, cutback of energy consumption during the synthesis of the binding material, reduction of CO_2_ emission by using less CaCO_3_ in the raw meal, and consumption of carbon dioxide. In the study, the kinetics of two-step synthesis of wollastonite from CaO and AlF_3_ production waste, namely, silica gel, its carbonisation process and the mechanical properties of obtained samples were investigated. According to XRD and DSC data, the optimal temperature in the mixture with CaO/(Al_2_O_3_ + SiO_2_) = 1 for the hydrothermal synthesis of the wollastonite precursors is 130 °C: F^−^–containing compounds were bound into katoite and cuspidine, and portlandite reacted completely within 8 h. The optimal temperature for wollastonite formation is 900 °C, but fluormayenite, cuspidine, and the traces of larnite form as well. During the curing in the CO_2_ atmosphere, wollastonite and larnite reacted completely and formed calcite, vaterite, and amorphous CaCO_3_. Cuspidine also participates in the carbonisation process and, in addition to amorphous SiO_2,_ it releases fluorite, which contributes to the total compressive strength of the products. The values of the compressive strength (10–15 MPa) in the wollastonite-sand samples match the requirements for the belite and special low-heat cements.

## Introduction

Wollastonite – calcium silicate CaSiO_3_, natural or artificial material, is widely used in ceramics, plastics, paints, paper industries and medicine fields. There are many ways to synthesise wollastonite, yet two of them are most common. The first is a single step synthesis, when limestone or other calcium source is mixed with silicon dioxide source (e. g. quartz) and then calcined in a temperature range of 1200–1400 °C to obtain wollastonite through solid state reactions^1–3^. The other way is a two-step synthesis, when calcium oxide and silicon dioxide are mixed with water to obtain suspension and then hydrothermally cured at 130–220 °C to obtain calcium silicate hydrates such as xonotlite, 1.13 nm tobermorite, and others^4,5^. It is also possible to synthesise these compounds by precipitation using calcium nitrate and sodium silicate solutions^6,7^. The obtained precursor is calcined at 800–900 °C when it recrystallizes into wollastonite^4–7^. This mineral has a wide range of applications in ceramics, cement, paints, biomedicine, etc. because of its desirable mechanical and chemical properties: low shrinkage, thermal stability, whiteness, hardness, and others^8–10^. When using amorphous silica instead of crystalline silicon for the initial mixture, calcium silicate hydrates are easily formed. However, the latter modification of SiO_2_ is quite rare in the nature, therefore, it is needed to search for alternative sources. One of them may be industrial waste such as AlF_3_ manufacturing by-product – silica gel.

While the fluorine-rich silica-gel waste is stored in landfills, the fluorine compounds can dissolve in the rainwater, leak and pollute the ground water. To prevent leakage, the fluorine can be bound into stable compounds. Silica-gel waste can be used as a source of silicon dioxide for the synthesis of calcium silicate hydrates^11–16^. This reaction is carried out in hydrothermal conditions and can also bound the pollutants of the silica-gel into stable calcium fluoride and calcium-silicate-aluminate compounds. As calcium fluoride is thermally stable, the products of the hydrothermal synthesis can be calcined to recrystallize calcium silicate hydrates into wollastonite^15,16^. The temperature of this process is ~900 °C, which is much lower than the temperature required for the single-step synthesis of wollastonite. Therefore, not only the energy demand but also CO_2_ emission is reduced because of lower fuel consumption.

The reduction of CO_2_ emission is a great challenge for the industry of binding materials as, during the production of ordinary Portland cement (OPC), ~5% of all man-made CO_2_ is emitted^17,18^. An alternative to the OPC could be low lime calcium silicates such as wollastonite CaSiO_3_ or rankinite Ca_3_Si_2_O_7_ ^19,20^. Even though wollastonite and rankinite are non-hydraulic, they can be activated by CO_2_ with the presence of humidity^21,22^. Wollastonite is more preferred for this process because of its better solubility in water, which means better reaction with CO_2_ and lower carbon dioxide emission caused by CaO production from CaCO_3_ ^23^. A newly formed matrix after the carbonation is composed of calcite CaCO_3_ and amorphous SiO_2_ provides similar mechanical properties of hydrated OPC where matrix consists mainly of calcium silicate hydrates and calcium hydroxide^21,24^.

Bukowski and Berger^24^ investigated mechanical properties of a binder which contained 50% sand and 50% wollastonite. They determined that the binder reached a compressive strength of 35 MPa within 24 h in 1 bar of CO_2_ using dynamic 1.4 *l*/min gas flow system (from the formation of the sample to the end of carbonisation). Other researchers^21^ reached a compressive strength of 70 MPa when the samples contained only wollastonite and were carbonised for 65 h at 60 °C in 100% carbon dioxide. This proves that wollastonite can be an alternative to OPC. Another advantage of wollastonite over ordinary Portland cement is that wollastonite can be synthesised from the same raw materials as the cement clinker and at 250–500 °C lower temperature^19,20,25^.

The aim of this work was to investigate the kinetics of a two-step synthesis of wollastonite from CaO and silica-gel waste, its carbonisation process, and the mechanical properties of the obtained samples. This work combines four aspects of eco-friendliness: (1) consumption of toxic waste, (2) cutback of energy consumption during the synthesis of the binding material, (3) reduction of carbon dioxide emission by using less CaCO_3_ in the raw meal, and (4) consumption of carbon dioxide.

## Materials and Methods

### Materials

AlF_3_ production by-product – silica gel from chemical plant SC Lifosa (Lithuania), dried at room temperature until constant mass for 2 weeks was used. The main elements were 39.86 wt% Si, 5.37 wt% Al (determined using X-ray fluorescence analyser Bruker X-ray S8 Tiger WD (Germany)), and 8.76 wt% F^−^ (determined potentiometrically using Mettler Toledo titrator T 70 (USA) with F^−^ selective electrode), mass losses – 4.1 wt% at 105 °C and 20.0 wt% at 1000 °C. The silica gel waste was milled for 2.5 min at 950 rpm using a planetary mill Fritsch Pulverisette 9 (Germany) until specific surface area *S*_*a*_ = 1537 m^2^/kg by Cilas LD 1090 (France) granulometer and density *ρ* = 2354 kg/m^3^ (gas pycnometer Quantachrome Instruments Ultrapyc 1200e, USA). Calcium oxide was obtained by calcining calcium hydroxide (≥96%, Honeywell, Germany) at 550 °C for 1 h and milling for 0.5 min at 950 rpm (*S*_*a*_ = 2076 m^2^/kg, *ρ* = 2837 kg/m^3^, free CaO – 94.22%). CEN standard sand (DIN EN 196-1 (ISO 679)) was used to press wollastonite-sand samples.

**Figure 1:**
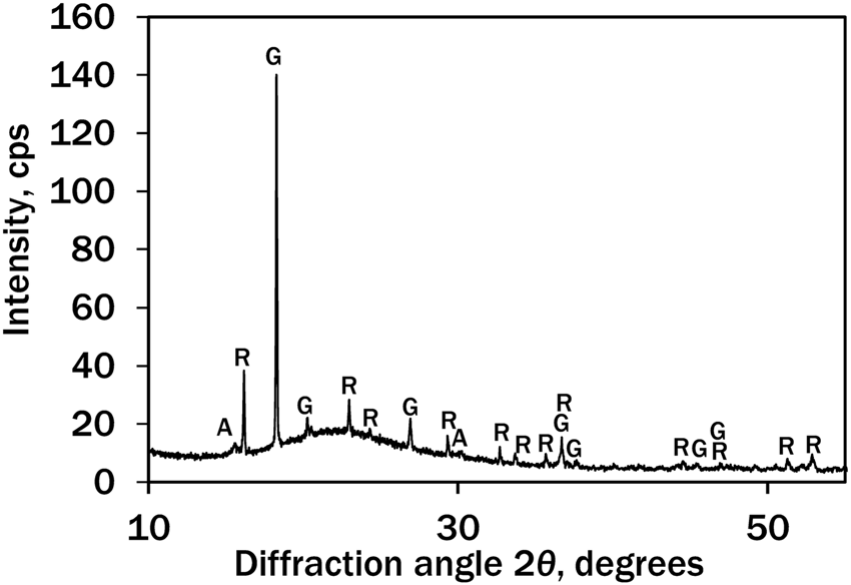
X-ray pattern of silica-gel waste. Indices: A – aliuminium hydroxyfluoride, R – rosenbergite, G – gibbsite.

Figure 1 shows that the silica-gel waste contains several crystalline compounds: aluminium hydroxyfluoride (Al_2_(OH)_3_F_3_·0.75H_2_O, PDF No. 74-0940, *d* = 0.5675, 0.2964, 0.2837 nm), rosenbergite (AlF_3_·3H_2_O, PDF No. 35-0827, *d* = 0.5460, 0.3860, 0.3299 nm) and gibbsite (Al(OH)_3_, PDF Nr. 07-324, *d* = 0.4850, 0.437, 0.4320 nm).

### Methods

The mixture of silica gel and calcium oxide with molar ratio CaO/(Al_2_O_3_ + SiO_2_) = 1 (48.31 wt% CaO, 42.39 wt% SiO_2_, 4.98 wt% Al_2_O_3_) was homogenised in Turbula Type T2F (Switzerland) for 1 h at 49 rpm and mixed with the distilled water to obtain the suspension with water/solid ratio W/S = 20. The hydrothermal syntheses were carried out in stirred suspensions (50 rpm) in Parr Instrument 4560 (USA) 600 mL autoclave at 95 and 130 °C, for 0–48 h. The products were filtered, rinsed with acetone to reduce carbonisation, and dried at 80 ± 0.3 °C for 24 h. The calcinations were carried out in the temperature range of 850–1050 °C (at the interval of 50 °C) for 1 h in Nabertherm B 180 (Germany) furnace. The calcined product-sand mixtures which contained 20%, 25, 30, and 35% calcined product were prepared. They were homogenised for 1 h at 49 rpm in Turbula Type T2F and mixed with water to obtain water/binding material ratio W/C = 0.35. The samples of 36 × 36 mm were pressed in a cylindrical frame under the pressure of 10, 12.5 and 15 kN at 1 kN/s speed and 20 s exposure at the maximum pressure in a Test Form Mega 10-400-50 (Germany) hydraulic press. The samples of humidified binding material without sand were also formed to determine the changes in the mineral composition during the curing process. The curing in the CO_2_ environment was carried out in Parr Instruments 4600 autoclave at 45 °C and 15 bar for 8-24 h. Before curing, the autoclave was purged twice from atmospheric air by letting CO_2_ up to 2 bar. The compressive strength was determined right after the curing using a Test Form Mega 10-400-50 hydraulic press at a loading rate of 1.5 kN/s. For the statistical accuracy, at least three samples of each batch were tested and the average value of a compressive strength was used.

The XRD analysis of dry powders was performed using D8 Advance diffractometer (Bruker AXS, Germany) operating at the tube voltage of 40 kV and tube current of 40 mA. The X-ray beam was filtered with 0.02 mm Ni filter to select the CuK_α_ wavelength. The diffraction patterns were recorded in a Bragg–Brentano geometry using a fast counting detector Bruker LynxEye based on a silicon strip technology. The specimens were scanned over the range 2*θ* = 3–70 at a scanning speed of 6 min^−1^ using a coupled two theta/theta scan type^26^.

The simultaneous thermal analysis (STA) was employed for the measuring phase transformation of the synthesised products at a heating rate of 10 °C/min, the temperature ranged from 30 to 945 °C in the nitrogen atmosphere, the mass of the sample was 20 mg. The test was carried out on a Linseis PT 1000 (Germany) instrument. The ceramic sample handlers and crucibles of Pt/10 wt%Rh were used^26^.

The amount of free CaO was determined according to standard ASTM C114–11b.

The quantitative analysis (QXRD) of the phases in the samples was carried out with the Rietveld method (Topas Software) by using XRD data.

BET analysis was performed by surface area analyser “Autosorb iQ Station 1” (Quantachrome Instruments, USA). The specific surface area of the hydrothermal synthesis products was calculated by the BET Eq. (1) using the data of the lower part of N_2_ adsorption isotherm (0.05 < $\frac{p}{{p}_{0}}$ < 0.35):

1 $$\frac{1}{X(\frac{{p}_{0}}{p}-1)}=\frac{C-1}{{X}_{m}\cdot C}\cdot \frac{p}{{p}_{0}}+\frac{1}{{X}_{m}\cdot C},$$

where *X* is the mass of adsorbate, adsorbed on the sample at relative pressure $\frac{p}{{p}_{0}}$, *p* the partial pressure of adsorbate, *p*_0_ the saturated vapour pressure of adsorbate, *X*_*m*_ the mass of adsorbate adsorbed at a coverage of one monolayer, *C* is a constant which is a function of the heat of the adsorbate condensation and heat of adsorption^27,28^.

When $\frac{1}{X(\frac{{p}_{0}}{p}-1)}-\frac{p}{{p}_{0}}$ is plotted versus $\frac{p}{{p}_{0}}$ (usually in the $\frac{p}{{p}_{0}}$ range of 0.05–0.35), BET equation yields a straight line. The slope coefficient $\frac{C-1}{{X}_{m}\cdot C}$ and the intercept $\frac{1}{{X}_{m}\cdot C}$ were used to determine *X*_*m*_ (2): when $S=\frac{C-1}{{X}_{m}\cdot C}$ and $I=\frac{1}{{X}_{m}\cdot C}$, then^27,28:^

2 $${X}_{m}=\frac{1}{S+I}.$$

## Results and Discussion

### Synthesis and calcination

**Figure 2:**
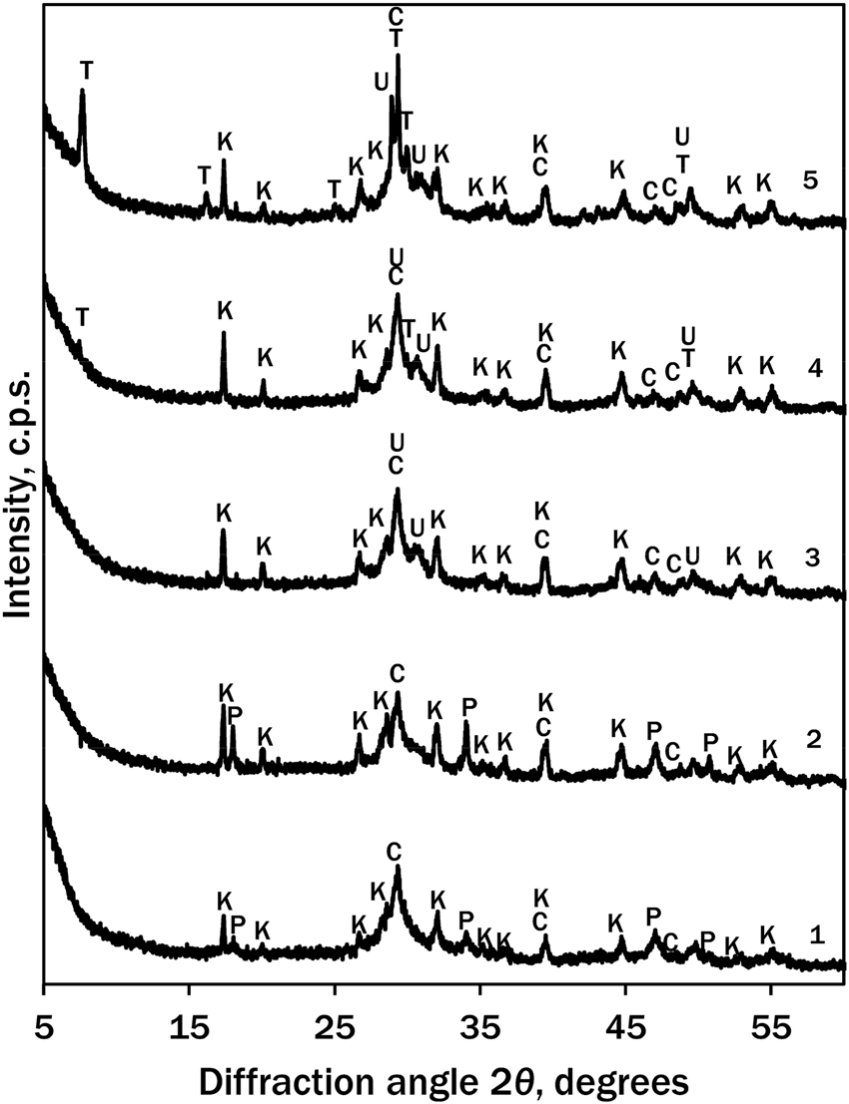
XRD patterns of hydrothermal synthesis products: 1–95 °C, 48 h; 2–130 °C, 4 h; 3–130 °C, 8 h; 4–130 °C, 24 h, 5–130 °C, 48 h. Indices: P – portlandite, K – katoite, C – calcite, U – cuspidine, T – 1.13 nm tobermorite.

According to XRD data, it was determined that during the isothermal curing at 95 °C some amount of portlandite Ca(OH)_2_ (PDF No. 04-0733; *d* = 0.2628; 0.4900; 0.1927 nm) remained unreacted even after 48 h (Fig. 2, curve 1).

The new crystalline compounds were katoite Ca_3_Al_2_(OH)_12_, (PDF No. 24-0217, *d* = 0.2295; 0.2039; 0.5130; 0.2810; 0.3358 nm) and calcite CaCO_3_, (PDF No. 07-8659; *d* = 0.3035; 0.1875; 0.2284 nm) which formed because of adsorption of atmospheric CO_2_. As portlandite was not completely bound even after 48 h at 95 °C, this temperature was considered not high enough and the same experiment was carried out in the increased temperature.

The reaction at 130 °C between CaO and SiO_2_ was much faster: even though portlandite was identified in the XRD pattern after 4 h (Fig. 2, curve 2), it was bound completely within 8 h (Fig. 2, curve 3). The new compounds after 4 h were the same as at 95 °C: katoite and calcite, but the intensity of their peaks increased. After 8 h cuspidine Ca_4_Si_2_O_7_F_2_ (PDF No. 76-0624, *d* = 0.3056; 0.2874; 0.2898; 0.3263; 0.2890 nm) was identified (Fig. 2, curve 3). It was determined that after 24 h (Fig. 2, curve 4) 1.13 nm tobermorite Ca_5_Si_6_O_16_(OH)_2_·4H_2_O (PDF No. 19-1364, *d* = 0.3080; 1.1300; 0.2980; 0.2820; 0.1842 nm) started to crystallise. The main crystalline compounds of hydrothermal synthesis products remained the same after 48 h of synthesis, but the amount and crystallinity of 1.13 nm tobermorite increased (Fig. 2, curve 5).

Since all portlandite was bound completely during the synthesis at 130 °C within 8 h and 1.13 nm tobermorite started to crystallise after 24 h, it was expected that this temperature would be more favourable rather than 95 °C for the hydrothermal synthesis of the precursors for wollastonite.

The admixtures in silica gel waste, i.e. aluminium fluoride and hydroxide, were bound into chemically inert compounds: katoite and cuspidine. The latter one is stable in the temperature range 20–1410 °C.

**Figure 3:**
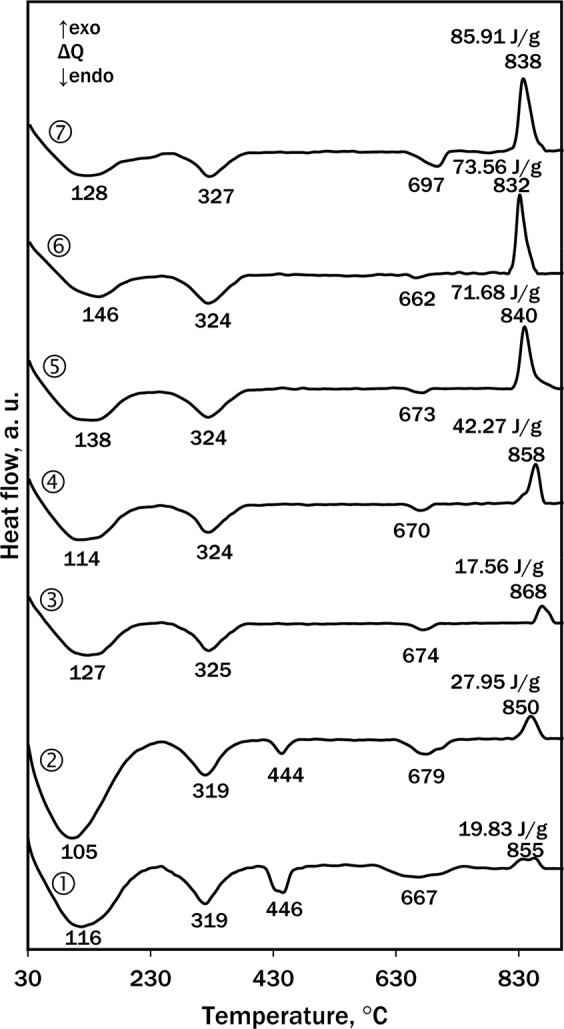
DSC curves of the hydrothermal synthesis at 130 °C products after: 1–0 h, 2–4 h, 3–8 h, 4–12 h, 5–24 h, 6–36 h and 7–48 h.

DSC results (Fig. 3) supplemented and confirmed the XRD data: after 0 h at 130 °C (Fig. 3 curve 1), the first endothermal effect in the DSC curve (100–130 °C) can be assigned to the loss of crystallisation water from calcium silicate hydrates, the second endothermal effect (320–325 °C) is typical to the decomposition of katoite, and the third endothermal effect (444–446 °C) is common to the dehydration of portlandite. The exothermal effect (832–868 °C) is typical to recrystallization of semi-crystalline C–S–H into wollastonite. The endothermal effect (662–697 °C) belongs to the decarbonisation of calcite. Both DSC and XRD showed that portlandite was bound within 8 h (Fig. 3 curve 3) because there was no endothermal effect at ~445 °C and no peak at 0.2627 nm in the XRD patterns.

The other important difference between the products shows the exothermal effect (830–870 °C) in the DSC curves. With a longer isothermal treatment duration, the effect migrates from the higher (850–870 °C after 0–12 h (Fig. 3, curves 1–4)) to the lower (840–832 °C after 24–48 h (Fig. 3 curves 5–7)) temperature and its enthalpy increases from ~20 to 86 J/g. This can be explained by different types of semi-crystalline C-S-H in the synthesis products: a small and “blunt” effect in samples of 0–12 h is common to C-S-H(II)^29^, while a tall and “sharp” one in the samples of 24–48 h belongs to C-S-H(I)^16,29^ which has lower basicity and is closer to the stoichiometric composition of the primary mixture.

The samples of hydrothermal synthesis at 130 °C (8, 24 and 48 h) were characterised by BET method (Table 1).

**Table 1:** Surface area and monolayer capacity of the samples of hydrothermal synthesis at 130 °C, determined by BET analysis.

Duration of hydrothermal synthesis, h	Sample mass, g	BET equation constants		Capacity of monolayer *X*_*m*_, g	*C*_*BET*_ constant	*S*_*BET*_, m^2^/g	*R*^2^
		Slope *S*	Intercept *I*				
8	0.0839	49.703	5.441 · 10^−1^	0.0197	92.356	69.308	0.99995
24	0.105	38.024	3.197 · 10^−1^	0.0256	119.937	90.824	0.99997
48	0.1486	41.77	3.588 · 10^−1^	0.0234	117.429	82.664	0.99997

It was determined that the surface area depended on the chemical/mineral composition of the formed compounds during hydrothermal synthesis. As it was mentioned, after 8–48 h of synthesis semi-crystalline C–S–H (II) (Fig. 3, curve 3, “blunt” exothermal effect at 868 °C), C–S–H (I) (Fig. 3, curve 5, “sharp” exothermal effect at 840 °C) and 1.13 nm tobermorite (Fig. 2, curve 4) were identified. For this reason *S*_*BET*_ values increased from 69.3 (8 h) to 90.8 m^2^/g (24 h). During 48 h of synthesis more 1.13 nm tobermorite (Fig. 2, curve 5) formed and its crystals were larger, therefore *S*_*BET*_ values of the sample decreased to 82.7 m^2^/g.

The products of hydrothermal syntheses were calcined in the temperature range of 850–1050 °C for 1 h to investigate the formation of wollastonite and the changes of its crystallinity.

**Figure 4:**
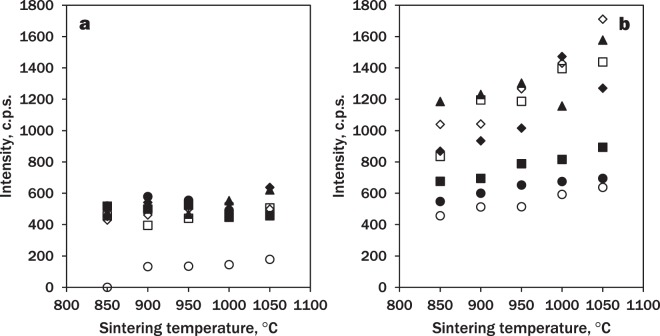
Intensity of the main peak of wollastonite (*d* = 0.2980 nm) in XRD patterns of calcined products. Hydrothermal synthesis temperature: a – 95 °C, b – 130 °C, duration: ○ – 0 h; ● – 4 h, ■ – 8 h, ◆ – 12 h, □ – 24 h, ◊ – 36 h, ▲ – 48 h.

**Figure 5:**
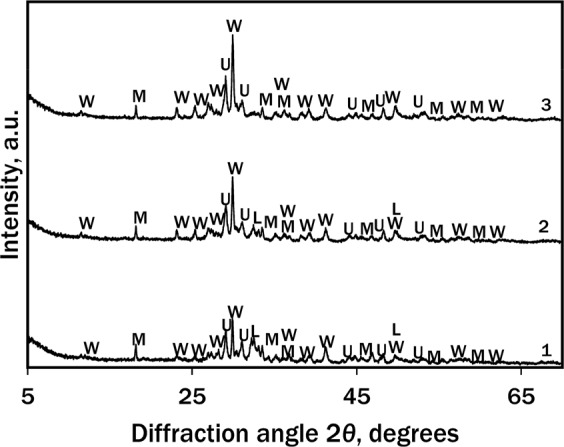
XRD patterns of calcined (900 °C 1 h) hydrothermal synthesis (130 °C) products after: 1–4 h; 2–12 h and 3–48 h. Indices: W – wollastonite; U – cuspidine; L – larnite; M – fluormayenite.

Even though the amount of portlandite in the products of hydrothermal synthesis at 95 °C decreased from 23.31% (0 h) to 4.73% (48 h), the intensity of wollastonite main peak (PDF No. 42–0550, *d* = 0.2980 nm) in the XRD patterns of calcined products (Fig. 4a) was in the range of 0–600 cps and did not depend neither on the hydrothermal synthesis duration (from 0 to 48 h) nor the calcination temperature (from 850 to 1050 °C). The amount of wollastonite in the calcined products of the hydrothermal synthesis at 130 °C (Fig. 4b) was much larger: the intensity range of the main peak of wollastonite was from 450 to 1750 cps. The products that contained portlandite (0–4 h) showed similar intensity (450–600 cps) of the wollastonite main peak to those of 95 °C synthesis (Fig. 5, curves 1). The products without portlandite had more intense peaks (600–1750 cps) of wollastonite and depended on both the synthesis duration and the calcination temperature. The latter dependence is more evident for the products of 12 h and longer syntheses (Fig. 5, curves 2 and 3). According to the intensity of the main peak of wollastonite in the XRD patterns, it can be concluded that portlandite had to react completely during the synthesis. As a result, after calcination, the products without portlandite contained more wollastonite, while the products with portlandite yielded this mineral less and contained more larnite Ca_2_SiO_4_ (PDF No. 33-0302; *d* = 0.2780; 0.2745; 0.2788; 0.2735; 0.2620 nm) – a compound where CaO/SiO_2_ molar ratio is twice higher than of the primary mixture.

According to the XRD data of the calcined samples, in addition to wollastonite and larnite, cuspidine and fluormayenite Ca_12_Al_14_O_32_F_2_ (PDF No. 87-2492; *d* = 0.4887; 0.2677; 0.2992; 0.2443; 0.2185 nm) were identified. The latter crystalline compounds are non-toxic, chemically and thermally stable up to 1000 °C.

**Figure 6:**
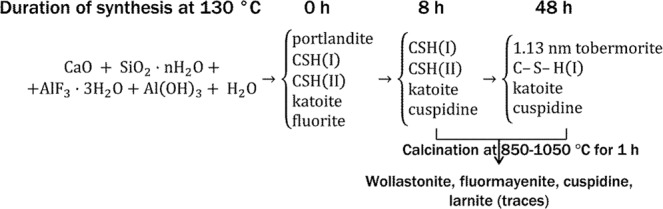
The summary of chemical processes in the CaO-silica-gel waste-water system during hydrothermal treatment at 130 °C and calcination in 850–1050 °C temperature range.

According to XRD and DSC data, the mineralogical composition variation of the products during the hydrothermal synthesis at 130 °C and calcination at 850–1050 °C was summarised in Fig. 6.

### Curing in the CO_2_ atmosphere

**Figure 7:**
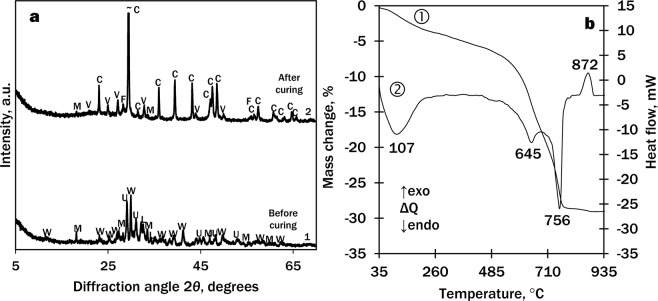
XRD patterns (**a**) and STA curves (**b**; 1 – TG, 2 – DSC) of the binding material after curing for 24 h at 45 °C and 15 bar CO_2_ (without sand, powdered). Indices: W – wollastonite; U – cuspidine; L – larnite; M – fluormayenite; C – calcite; V – vaterite; F – fluorite.

The product of the hydrothermal synthesis at 130 °C for 8 h (Fig. 2, curve 3) and calcining at 900 °C for 1 h (Fig. 7a, curve 1) was used for the production of wollastonite-based mortar samples and their potential for hardening in the CO_2_ environment was determined. The mineral composition of the binding material is given in Table 2.

**Table 2:** Mineral composition of the binding material according to QXRD analysis.

Compound	Formula	Amount
Wollastonite	CaSiO_3_	32.08%
Larnite	Ca_2_SiO_4_	32.29%
Cuspidine	Ca_4_Si_2_O_7_F_2_	26.50%
Fluormayenite	Ca_12_Al_14_O_32_F_2_	9.13%

These conditions were chosen from the economic point of view because 8 h was the shortest period of time for the hydrothermal synthesis to bind portlandite completely and the calcination at 900 °C for 1 h is sufficient to produce wollastonite.

**Figure 8:**
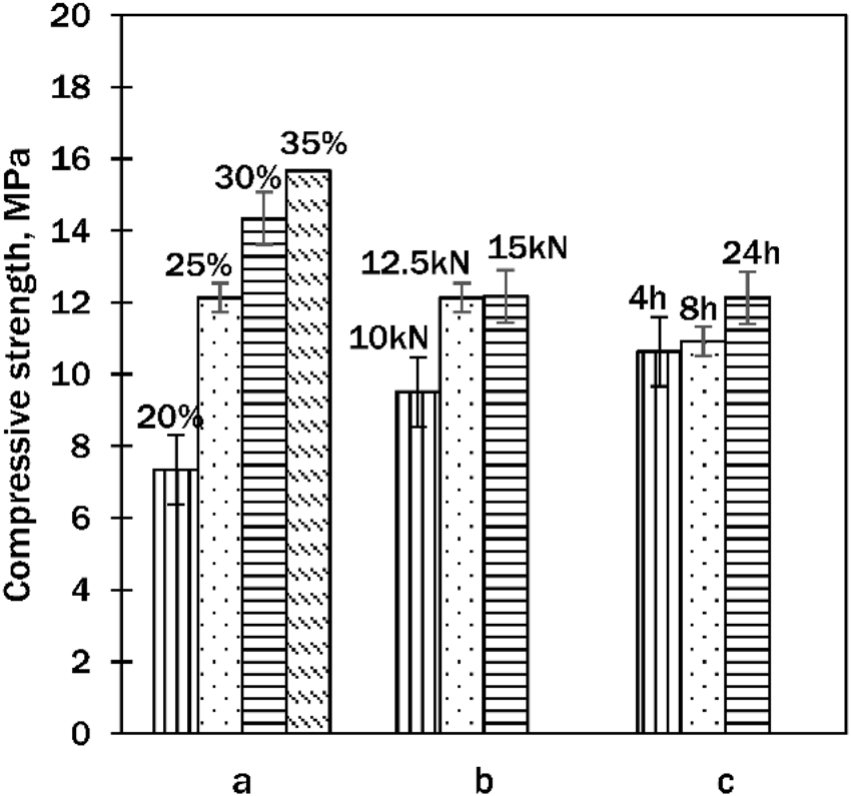
Compressive strength of cured samples dependency on: (**a**) – amount of binding material, (**b**) – compaction pressure, (**c**) – curing duration. (**a**,**b** – cured for 24 h; **b,c** – 25 wt% of binding material).

It was determined that the compressive strength depended mostly on the amount of binding material in the samples. By increasing the binding material from 20 to 35 wt%, the strength boosted more than twice: from 7.34 to 15.68 MPa (Fig. 8a). With the increment of compaction pressure, when the binder/sand ratio was 1:3 (amount of binder – 25 wt%), the compressive strength went up by a third: from 9.51 (10 kN) to 12.15 (15 kN) MPa (Fig. 8b). The curing duration had a low impact on the compressive strength, as the increase of compressive strength was only 14%: from 10.64 (4 h) to 12.13 (24 h) MPa (Fig. 8c).

It was determined that the main compounds in the binding material before curing (Fig. 7a, curve 1) were wollastonite CaSiO_3_ (PDF No. 42-0550; *d* = 0.2981; 0.3323; 0.3089; 0.3519; 0.3843 nm), cuspidine, and fluormayenite with some traces of larnite.

After curing for 24 h at 45 °C and 15 bar CO_2_ (Fig. 7a, curve 2), the main components were two polymorphs of calcium carbonate CaCO_3_: calcite (PDF No. 07-8659, *d* = 0.3035; 0.1875; 0.2284; 0.1912; 0.2094 nm) and vaterite (PDF No. 67-0200, *d* = 0.2721; 0.3292; 0.1820; 0.4229; 0.3574 nm) with small amounts of fluormayenite and fluorite CaF_2_ (PDF No. 01-1274, *d* = 0,1932; 0,3155; 0,1647 nm). Wollastonite, larnite and cuspidine reacted completely. According to Ashraf *et al*.^21^, during the carbonation reaction of wollastonite, XRD-amorphous SiO_2_ is released.

According to the STA data (Fig. 7b), the first endothermal effect in the DSC curve (107 °C) is typical to the loss of adsorbed water, and the second double endothermal effect (640–760 °C) is typical to the decomposition of carbonates: the first one (645 °C) – decarbonisation of vaterite and the second one (756 °C) – calcite. This suggests that very low onset temperature (~500 °C) may be related to the decomposition of amorphous calcium carbonate so these results are directly in line with previous findings^22,30^. To describe the origin of the exothermal effect at 872 °C, the sample was calcined at 820 and 900 °C. According to the XRD patterns, the effect belongs to the formation of wollastonite from calcium oxide, a product of calcination of CaCO_3_, and amorphous SiO_2_, which was released during carbonation process.

The degree of carbonation was calculated from the TG data of the samples without sand (W/C = 0.35, compaction pressure 12.5 kN, curing duration: 4, 8 and 24 h) and it was determined that in all the samples without sand were ~23 wt% CO_2_. The maximum amount of CO_2_ that can be stored in the binding material can be calculated using Steinour formula (3)^31,32^:

3 $${{\rm{C}}{\rm{O}}}_{2}({\rm{ \% }}\,max)=0.785({\rm{C}}{\rm{a}}{\rm{O}}-0.7{{\rm{S}}{\rm{O}}}_{3})+1.091{\rm{M}}{\rm{g}}{\rm{O}}+1.420{{\rm{N}}{\rm{a}}}_{2}{\rm{O}}+0.935{{\rm{K}}}_{2}{\rm{O}},$$

here: CaO, SO_3_, MgO, Na_2_O, and K_2_O are the mass percentages of relevant constituent oxides. In our case, in the initial mixture, there are only CaO = 48.31 wt%. So:

$${{\rm{CO}}}_{2}( \% \,{\rm{\max }})=0.785\,\cdot \,48.31 \% =37.92 \% .$$

37.92 wt% of CO_2_ corresponds to 86.19 wt% of CaCO_3_ and 23 wt% – to 52.27 wt%.

To calculate the degree of carbonation formula (4)^21^ was used:

4 $${\rm{Degree}}\,{\rm{of}}\,{\rm{carbonation}}, \% =\frac{{\rm{Amount}}\,{\rm{of}}\,{{\rm{CaCO}}}_{3}\,(\mathrm{wt} \% )}{{\rm{Maximum}}\,{\rm{amount}}\,{\rm{of}}\,{{\rm{CaCO}}}_{3}\,(\mathrm{wt} \% )}\times 100 \% ;$$

$${\rm{Degree}}\,{\rm{of}}\,{\rm{carbonation}}, \% =\frac{52.27 \% }{86.19 \% }\times 100 \% =60.64 \% .$$

### Investigation of CO_2_ curing of cuspidine

**Figure 9:**
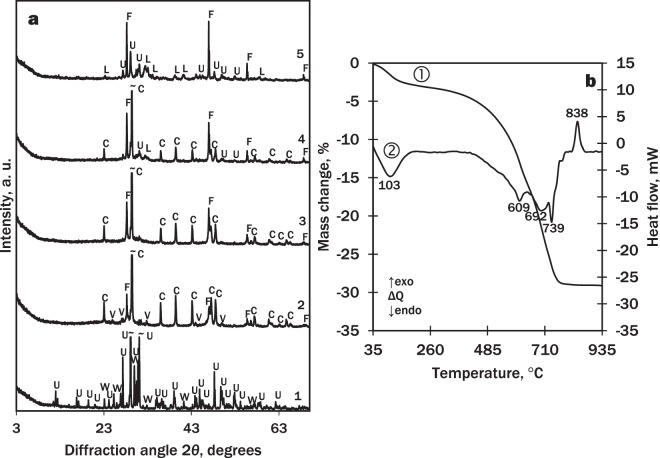
(**a**) X-ray diffraction patterns of cuspidine samples: 1 – before curing; 2 – after curing for 24 h at 15 bar CO_2_ in 45 °C; 3 – cured sample fired at 622 °C; 4 – cured sample fired at 724 °C, 5 – cured sample fired at 790 °C, (**b**) STA curves (1 – TG, 2 – DSC) of carbonated (24 h, 15 bar, 45 °C) cuspidine. Indices: U – cuspidine, W – wollastonite, C – calcite, V – vaterite, F – fluorite, L – larnite.

According to the XRD data, the products of calcination along with wollastonite always contained cuspidine Ca_4_Si_2_O_7_F_2_ which forms from admixtures of silica-gel waste and calcium oxide. However, the literature sources lack data describing the role of cuspidine in the CO_2_-curing process. To investigate what chemical changes the mineral undergoes during the curing in the CO_2_ atmosphere, cuspidine was synthesised from reagent grade materials using the hydrothermal synthesis at 130 °C for 24 h and calcination at 900 °C for 1 h) (Fig. 9a, curve 1). Sample preparation (water/solid ratio = 0.35, compaction pressure – 12.5 kN) and curing (for 24 h in 15 bar CO_2_ at 45 °C) conditions were the same as in the wollastonite case. According to the XRD data, the cured sample no longer contained cuspidine, however, there were fluorite and two types of calcium carbonate – calcite and vaterite, instead (Fig. 9a, curve 2).

The STA results confirmed the XRD data (Fig. 9b). The DSC curve showed four endothermal effects: the first one at 103 °C is typical to loss of adsorbed water, and the other three occurred at 609, 692 and 739 °C. To determine the latter effects, the samples of cured cuspidine were calcined at corresponding temperatures. According to the XRD data (Fig. 9a, curve 3), the effect at 609 °C means the decomposition of vaterite and the effects at 692 and 739 °C – double decomposition of calcite (Fig. 9a, curves 4 and 5). The very low onset temperature (~500 °C) may be related to the decomposition of amorphous calcium carbonate similarly to^22,30^. The exothermal effect at 838 °C can be attributed to the formation of calcium silicates. In conclusion, the processes during the calcination of cured wollastonite and cuspidine are similar.

According to the obtained data, the chemical equation of carbonisation of cuspidine may be written by formula (5):

5 $${{\rm{Ca}}}_{4}{{\rm{Si}}}_{2}{{\rm{O}}}_{7}{{\rm{F}}}_{2}+3{{\rm{CO}}}_{2}+{{\rm{nH}}}_{2}{\rm{O}}\to 3{{\rm{CaCO}}}_{3}+{{\rm{CaF}}}_{2}+2{{\rm{SiO}}}_{2}\,\cdot \,{{\rm{nH}}}_{2}{\rm{O}},$$

Therefore, cuspidine, analogically to wollastonite, also reacts with carbon dioxide and forms calcite, which contributes to the total compressive strength of the cured products. However, in addition to calcium carbonate, inert compounds (in this system^33^), namely, amorphous silica and fluorite are released. According to the Mohs’ scale, the latter mineral is harder than calcite^34^.

To conclude the investigation of cuspidine curing in the elevated CO_2_ pressure, it should be pointed out that silica-gel waste which contains fluoride ions can be used as a source of SiO_2_ for the wollastonite synthesis and as a binder for CO_2_-cured products, because the F^−^ ions are bound into cuspidine which participates in the carbonation process in the same way as calcium silicates.

## Conclusions

1. The study showed that AlF_3_ production waste, silica gel, is suitable for the wollastonite binder synthesis and, subsequently, could be used for the production of carbonated construction materials. This process is an environmentally-friendly approach reducing carbon footprint as well as the consumption of toxic waste. Furthermore, this could be an economically viable option as wollastonite binder production requires 550 °C lower temperature than Ordinary Portland cement.
2. It was determined that, in the CaO/SiO_2_ mixture, the optimal temperature for the hydrothermal synthesis of the precursors of wollastonite is 130 °C: the admixtures in silica gel (aluminium fluoride and hydroxide) were bound into chemically non-aggressive katoite and cuspidine and portlandite reacted completely within 8 h.
3. The precursors for the sintering of wollastonite must be Ca(OH)_2_–free, because the products with portlandite after the calcination contained less wollastonite and more larnite. The intensity of wollastonite peaks from precursors without portlandite was higher (600–1750 cps) and it augments with prolonging the hydrothermal synthesis duration and increment of calcination temperature. Fluormayenite, cuspidine and the traces of larnite forms as well.
4. During the curing calcined products in the CO_2_ atmosphere, wollastonite, larnite, and cuspidine reacted completely and formed several polymorphs of calcium carbonate: calcite, vaterite and amorphous CaCO_3_. It was determined that, during cuspidine carbonisation, in addition to amorphous silica, it releases fluorite. Fluormayenite remained inert during the curing process.
5. Wollastonite-sand samples gain relatively high compressive strength (10–15 MPa) during curing in the CO_2_ atmosphere. It was determined that with increment of the amount of binding material, compaction pressure, as well as the exposure duration in the CO_2_ environment, sample compressive strength is highly increased. These values meet the requirements for the belite and special low-heat cements.

## References

[1] Nour WMN, Mostafa AA, Ibrahim DM (2008). Recycled wastes as precursor for synthesizing wollastonite. Ceram. Int..

[2] Heriyanto PF, Sahajwalla V (2018). Synthesis of calcium silicate from selective thermal transformation of waste glass and waste shell. J. Clean. Prod..

[3] Saravanapavan P, Hench LL (2003). Mesoporous calcium silicate glasses. I. Synthesis. J. Non-Cryst. Solids..

[4] Ismail H, Shamsudin R, Hamid MAA (2016). Effect of autoclaving and sintering on the formation of β-wollastonite. Mater. Sci. Eng. C..

[5] Pei LZ (2010). A green and facile route to synthesize calcium silicate nanowires. Mater. Charact..

[6] Lin K, Chang J, Lu J (2006). Synthesis of wollastonite nanowires via hydrothermal microemulsion methods. Mater. Lett..

[7] Lin K, Chang J, Chen G, Ruan M, Ning C (2007). A simple method to synthesize single-crystalline β-wollastonite nanowires. J. Cryst. Growth..

[8] Saadaldin SA, Rizkalla AS (2014). Synthesis and characterization of wollastonite glass-ceramics for dental implant applications. Dent. Mater. J..

[9] Kalla P (2013). Mechanical and durability studies on concrete containing wollastonite–fly ash combination. Constr. Build. Mater..

[10] Lin K (2005). Study of the mechanical property and *in vitro* biocompatibility of CaSiO_3_ ceramics. Ceram. Int..

[11] Vaičiukynienė D (2015). Effects of ultrasonic treatment on zeolite NaA synthesized from by-product silica. Ultrason. Sonochem..

[12] Girskas G, Skripkiūnas G, Šahmenko G, Korjakins A (2016). Durability of concrete containing synthetic zeolite from aluminum fluoride production waste as a supplementary cementitious material. Constr. Build. Mater..

[13] Sarawade PB, Kim JK, Hilonga A, Kim HT (2010). Recovery of high surface area mesoporous silica from waste hexafluorosilicic acid (H_2_SiF_6_) of fertilizer industry. J. Hazard. Mater..

[14] Elineema G (2013). Quantitative recovery of high purity nanoporous silica from waste products of the phosphate fertilizer industry. J. Ind. Eng. Chem..

[15] Iljina A, Baltakys K, Bankauskaite A, Eisinas A, Kitrys S (2016). The stability of formed CaF_2_ and its influence on the thermal behavior of C–S–H in CaO–silica gel waste-H_2_O system. J. Therm. Anal. Calorim..

[16] Iljina A, Baltakys K, Bankauskaite A (2015). Thermal properties and application of silica gel waste contaminated with F− ions for C-S-H synthesis. J. Therm. Anal. Calorim..

[17] Humphreys, K. & Mahasenan, M. Toward a sustainable cement industry, Substudy 8. World Business Council for Sustainable Development. Internet access, https://www.wbcsdcement.org/pdf/battelle/final_report8.pdf (last accessed 05 March 2019).

[18] Scalenghe R (2011). Influence of 150 years of land use on anthropogenic and natural carbon stocks in Emilia-Romagna region (Italy). Environ. Sci. Technol..

[19] Kaifi FMZ, Aurangzeb M, Ahmed B, Khan M (2004). Manufacture of synthetic wollastonite. J. Chem. Soc. Pakistan..

[20] Smigelskyte A, Siauciunas R, Wagner M, Urbonas L (2019). Synthesis of rankinite from natural Ca-Si rocks and its hardening in CO_2_ atmosphere. Rev. Rom. Mater..

[21] Ashraf W, Olek J, Tian N (2016). Multiscale characterization of carbonated wollastonite paste and application of homogenization schemes to predict its effective elastic modulus. Cement Concrete Comp..

[22] Ashraf W, Olek J (2016). Carbonation behavior of hydraulic and non-hydraulic calcium silicates: potential of utilizing low-lime calcium silicates in cement based materials. J. Mater. Sci..

[23] Daval D (2009). Carbonation of Ca-bearing silicates, the case of wollastonite: experimental investigations and kinetic modelling. Chem Geol..

[24] Bukowski JM, Berger RL (1978). Reactivity and strength development of CO_2_ activated non-hydraulic calcium silicates. Cement Concrete Res..

[25] Sahu, S. & DeCristofaro, N. Solidia Cement™, Solidia Technology. White Paper. 2013. Internet access, http://solidiatech.com/ (last accessed 05 March 2019).

[26] Baltakys K, Eisinas A, Doneliene J, Dambrauskas T, Sarapajevaite G (2019). The impact of Al_2_O_3_ amount on the synthesis of CASH samples and their influence on the early stage hydration of calcium aluminate cement. Ceram. Int..

[27] Eisinas A, Baltakys K, Siauciunas R (2012). The effect of gyrolite additive on the hydration properties of Portland cement. Cement Concrete Res..

[28] Dambrauskas T, Baltakys K, Eisinas A, Kitrys S (2019). The specific surface area and porosity of synthetic and calcined α-C_2_SH, kilchoanite and hydroxyledgrewite. Powder Technol..

[29] Rodriguez ET, Garbev K, Merz D, Black L, Richardson IG (2017). Thermal stability of C-S-H phases and applicability of Richardson and Groves’ and Richardson C-(A)-S-H(I) models to synthetic C-S-H. Cement Concrete Res..

[30] Huber M (2005). Flame synthesis of calcium carbonate nanoparticles. Chem. comm..

[31] Sugunan A, Kurian SS (2017). Non Uniform Corrosion of Carbon Cure RC Beam and Conventional RC Beam. Int. Res. J. Eng. Tech..

[32] Steinour HH (1959). Some effects of carbon dioxide on mortars and concrete discussion. J. Am. Concr. Inst..

[33] Aigueperse, J. *et al*. Fluorine Compounds, Inorganic in *Ullmann’s Encyclopedia of* Industrial Chemistry (ed. Ullman, F.) (Wiley-VCH, 2005), 10.1002/14356007.a11_307, ISBN 3527306730.

[34] Klein, C. Minerals and Rocks: Exercises in Crystallography, Mineralogy, and Hand Specimen Petrology (John Wiley & Sons, 1989).

